# “You Don’t Know If It’s the Truth or a Lie”: Exploring Human Papillomavirus (HPV) Vaccine Hesitancy among Communities with Low HPV Vaccine Uptake in Northern California

**DOI:** 10.3390/vaccines12040372

**Published:** 2024-04-01

**Authors:** Julie H. T. Dang, Alexandra Gori, Lucy Rios, Angelica M. Rolon, Jingwen Zhang, Moon S. Chen

**Affiliations:** 1 Division of Health Policy and Management, Department of Public Health Sciences, School of Medicine, University of California, Davis, CA 95816, USA; 2 Comprehensive Cancer Center, University of California, Davis, CA 95816, USA; acgori@ucdavis.edu (A.G.); amiperez@ucdavis.edu (A.M.R.); 3 Division of Hematology and Oncology, Department of Internal Medicine, School of Medicine, University of California, Davis, CA 95817, USA; lucrios@ucdavis.edu (L.R.); mschenjr@ucdavis.edu (M.S.C.J.); 4 Department of Public Health Sciences, University of California, Davis, CA 95817, USA; 5 Department of Communication, Department of Public Health Sciences, University of California, Davis, CA 95616, USA; jwzzhang@ucdavis.edu

**Keywords:** human papillomavirus (HPV) vaccine hesitancy, key informant interviews, focus groups, qualitative research, racially/ethnically diverse communities, rural

## Abstract

Background: Vaccine hesitancy, delaying or refusing to vaccinate despite the availability of vaccines, impedes the progress of achieving optimal HPV vaccine coverage. Little is known about the sources of human papillomavirus (HPV) vaccine hesitancy among racially/ethnically and geographically diverse communities. The purpose of this paper is to explore HPV vaccine hesitancy among rural, Slavic, and Latino communities that reside in counties with low HPV vaccine uptake rates. Methods: Key informant interviews and focus groups were conducted with rural, Slavic, and Latino communities that reside within counties in California that have low HPV vaccine up to date rates (16–25%). Qualitative data were transcribed verbatim and analyzed using inductive and deductive thematic analysis. Results: A total of seven focus groups and 14 key informant interviews were conducted with 39 individuals from seven California counties. Salient themes that contributed to HPV vaccine hesitancy included the following: social media and the anti-vaccination movement; a strong belief in acquiring immunity naturally; prior vaccine experiences; and vaccine timing concerns. Participants suggested the provision of culturally appropriate, in-language, in-person easy to understand HPV vaccine education to mitigate HPV vaccine hesitancy. Conclusions: Our findings can inform future interventions to increase HPV vaccine uptake among hesitant communities.

## 1. Introduction

The World Health Organization (WHO) considers vaccine hesitancy as one of the top threats to global health [1]. Vaccine hesitancy, defined as a “reluctance or refusal to vaccinate despite the availability of vaccines”, impedes the progress of achieving optimal HPV vaccine coverage [2]. While vaccine hesitancy is not a new phenomenon, greater access to and more rapid dissemination of vaccine misinformation via social media and the Internet, coupled with a lower prevalence of vaccine-preventable diseases, an extensive childhood vaccination schedule, and rising public skepticism, have all contributed to human papillomavirus (HPV) vaccine hesitancy [3,4]. Since its introduction in 2006, the HPV vaccine has experienced public distrust and criticism [5,6]. This hesitancy has been attributed to a lack of confidence in the vaccine’s safety, misinformation, perceived low risks of vaccine-preventable diseases, and perceptions that the vaccine does not work [3,7,8]. In recent years, deploying strategies to mitigate the effects of vaccine hesitancy has proven to be challenging, especially because of the COVID-19 pandemic. The pandemic created postponement of routine health care, including HPV vaccination [9], and ignited new vaccine hesitancies [10].

A study analyzing data from the CDC’s National Immunization Survey (NIS)-Teen Survey from 2012 to 2018 found that despite a provider recommendation, 60.6% of unvaccinated adolescents had no intention to initiate the HPV vaccine series [11]. Over the six-year study period, parental reluctance to initiate the HPV vaccine series for girls increased from 54.1% to 68.1%; and for boys, parental reluctance for the vaccine rose from 44.4% to 59.2% [11]. Despite the HPV vaccine being a highly effective public health intervention, in 2022, only 62.6% of U.S adolescents aged 13–17 were up to date with HPV vaccine (UTD HPV) [12], which continues to remain below the Healthy People 2030 goal of 80% [13]. While HPV vaccination rates have begun to steadily increase, studies have reported disparities in HPV vaccination rates and in HPV-associated cancers among rural communities [14,15,16] and among racial and ethnic groups [17,18]. The goal of this paper is to investigate sources of HPV vaccine hesitancy among rural, Latino, and Slavic communities. Additionally, we sought to gain insight from these individuals on strategies and recommendations to improve HPV vaccine acceptance in their respective communities.

## 2. Materials and Methods

### 2.1. Study Sample and Recruitment

We recruited parents, health professionals, and community members from rural inland northern California counties with lower UTD HPV rates than the U.S. (16–25% compared to 62.6%) [12,13,14,15,16,17,18,19] and from Latino and Slavic communities located in the University of California, Davis Comprehensive Cancer Center’s catchment area to participate in semi-structured focus groups and key informant interviews. We did not include the counties with the lowest HPV vaccination rates in the catchment area in this study as another study has been published that focused on those rural counties and Native American adolescents [20]. Participants were recruited from Nevada, Placer, Yolo, El Dorado, Sacramento, San Joaquin, and Merced Counties. Eligibility included being at least 18 years of age and residing or working in one of the above seven counties. We define rural as having a Rural-Urban Community Area Codes (RUCA) classification of 4–10 [21]. While all seven counties have RUCA zip codes that are designated as rural, for the purpose of our study, participants from Nevada and El Dorado Counties were considered rural. The majority of individuals who identify as Slavic are from countries located mostly in Eastern Europe and Western Asia and speak Polish, Russian, and Ukrainian. We purposively targeted Sacramento, Placer, and Yolo counties for Slavic participants as the majority reside there; and for Hispanic/Latino participants, we targeted the counties of San Joaquin and Merced. We utilized StudyPages, an online participant-facing platform, to recruit and register participants to the study [22]. Through flyers, listservs, outreach to past research participants, social media advertisement, engagement with community partners, and a media release, participants were directed to our StudyPages website to screen for study eligibility, register, provide language preference, and specify whether they wanted to participate in a focus group or interview. Eligible participants were contacted by the study team to coordinate a date and time.

As an alternative to StudyPages, the participants were also provided with the study team’s phone number and email for direct contact. Focus groups and interviews were conducted during the period of February 2021 to September 2021. Verbal informed consent from each participant was obtained at the beginning of each session and the participants received a USD 20 gift card for their time. Data collection continued until saturation was reached with no new themes emerging from additional interviews and or focus groups. Focus groups and interviews lasted between 30 and 60 min. This study was approved by the University of California, Davis Institutional Review Board.

### 2.2. Focus Group and Interview Guide

Focus groups and interviews were conducted in English, Spanish, Russian, and were conducted in person and virtually, using Zoom. Each focus group or interview was conducted by a trained facilitator and in the language of preference of the participant. Focus group and interview questions came from the Vaccine Hesitancy Determinants Matrix (VHDM) and a survey tool developed by the Strategic Advisory Group of Experts (SAGE) Working Group on Vaccine Hesitancy [2,23]. The matrix organizes factors that contribute to vaccine hesitancy into three main categories: contextual, individual and group, and vaccine/vaccination-specific influences. See Table 1 for determinants, constructs, and general focus/interview guide questions. In our introduction, we told participation we were interested in both their personal experiences and perspectives about general vaccinations and the human papillomavirus (HPV) vaccine. For participants who were not familiar with the HPV vaccine, we provided a brief explanation of the vaccine. To begin, we asked participants what their current thoughts were on vaccinations and if they were familiar with the HPV vaccine. Questions were tailored and adapted based on the participants’ familiarity of the HPV vaccine. Additionally, we asked all participants what they thought would be successful strategies that can help with increasing the uptake of the HPV vaccine within their community. 

### 2.3. Data Analysis

All focus groups and interviews were transcribed verbatim, uploaded onto Dedoose [24], and analyzed using inductive and deductive thematic analysis [25,26]. For the Spanish and Russian focus groups and interviews, the recordings were transcribed from the native language audio file to English by bilingual study team members. A team of three coders reviewed and analyzed all transcripts first using an inductive coding approach, allowing for themes and codes to emerge from the raw data. After this initial coding, a deductive approach was used to group similar themes and codes into categories based on the domains and constructs of the VHDM. The first author (principal investigator for the study) read all transcripts and randomly coded ten interviews to ensure consistency. The coders and the PI met weekly to review, reconcile, refine, and define themes, codes, and categories, and to resolve disagreements. Representative quotes were identified to support categories. After salient themes were determined, a post-study focus group was conducted with a group of prior participants to confirm preliminary data analysis. 

## 3. Results

A total of seven focus groups (five in English, one in Spanish, and one in Russian) and fourteen key informant interviews (nine in English, one in Spanish, and four in Russian) were conducted with 39 individuals from the Nevada (*n* = 7), Placer (*n* = 2), Yolo (*n* = 3), El Dorado (*n* = 2), Sacramento (*n* = 8), San Joaquin (*n* = 8), and Merced (*n* = 9) counties. Most participants were female (87.2%) and identified as being a parent (82.1%). About a quarter of the participants were from the Latino/Hispanic community (25.6%); 17.9% were from the rural non-Hispanic White community; 15.4% were from the Slavic community; a little over a third (35.9%) represented multiple communities (e.g., worked with all communities, Latino/Hispanic, and/or rural); and 5.1% represented other communities (e.g., urban non-Hispanic White, Hmong). See Table 2 for a description of the participants.

We organized our findings into the three categories of the Working Group on Vaccine Hesitancy Determinants Matrix, and within each category, we describe the themes we found to be the most salient. Under contextual influences, we report on the role of communication and media environment, as well as the antivaccination movement, in contributing to HPV vaccine hesitancy. The antivaccination movement can be explained as a combination of several determinants under contextual influences (e.g., anti-vaccination lobby, religion/culture, politics/policies, and perceptions of the pharmaceutical industry); thus, below, we have it listed as its own determinant. Under individual and group influences, we describe participants vaccination beliefs, and attitudes about health and prevention; risk perceptions; and how their personal, family and/or community members’ experience with vaccination are sources of HPV vaccine hesitancy. For vaccine/vaccination-specific influences, we discuss the participants’ views on the timing of administering the HPV vaccine. Since late 2016, only Garadisil-9 is distributed to the U.S.; thus, we assumed all discussions regarding the HPV vaccine were in reference to the 9-valent vaccine. 

### 3.1. Contextual Influences

All participants shared that contextual influences contributed to both their HPV vaccine and overall vaccine hesitancy. The research team identified two constructs within this determinant as themes that were most salient in the focus groups and interviews: (1) communication, and media environment; and (2) the anti-vaccination movement. 

#### 3.1.1. Communication and Media Environment

Social media coverage of the HPV vaccine was identified as a major source of HPV vaccine hesitancy among participants. Participants shared examples of how easy it is to post vaccine information (regardless of accuracy and validity) on social media platforms such as Facebook, Instagram, TikTok, and YouTube. One participant said, “On Facebook [you] can put any information out and anyone can say what they want, and no one can know if it’s the truth or lie” (Participant 1, Interview, Nevada County). When asked if they can recall having seen anything on social media that may have made them reconsider vaccinating their child against HPV, one participant recalled, “…on TikTok people really follow really quickly [in reference to how people can quickly amass followers and with videos going vial] then there could be a lot of misinformation through Tik Tok because anybody can post whatever and make it sound a certain way” (Participant 2, Focus Group #1, San Joaquin County). This impression of how effectively individuals can create and post sensualized HPV vaccine messaging on social media and the reoccurrence of these videos is explained by this participant:

*“If I’m watching some like YouTube thing like they’ll have some educational stuff on vaccines. But you look on the side of whatever you’re watching the next thing that pops up will be something like the signs of why you should not get the HPV vaccine. People easily get sucked into these videos and the videos will just play automatically. It’s like oh my gosh, this story is crazy and the only thing that happened was this vaccine. I think it’s these lived experience stories even if you don’t know the person or remotely know the person, it could be actors for all you know, but the story itself stands out”*

(Participant 5, Focus Group, Nevada County).

In terms of what they can remember reading and or seeing on social media, the majority reported that the information was always negative. Participants recalled that the main take-away messages from the vaccine information they have heard on social media focused on the notion that vaccines can cause autism, as well as an emphasis on severe and not necessarily correct adverse effects. One participant shared, “I have seen groups on Facebook of mothers that are against the vaccinations [HPV] and that also did say that it could cause autism and that it did certain things to their kids” (Participant 3, Focus Group #1, Merced County). Another participant disclosed, “I see a lot of people sharing [on YouTube] their vaccine horror stories of how bad their reaction was, or they know someone who had a horrible reaction and those are really easy to find and they’re the first things that pop up when you research vaccines” (Participant 4, Interview, El Dorado County). A participant shared that, similarly, she receives social media videos from her friends that spread HPV vaccine misinformation. She described a video in which the main point of the story was how a study came out in Spain that talked about how the HPV vaccine affected adolescents over there, she said “…that friend of mine [who shared the video] said, no don’t be putting the vaccine [HPV] to your kids, you are going to provoke a reaction or they become sterile” (Participant 1, Interview, Nevada County). 

#### 3.1.2. Anti-Vaccination Movement 

Participants discussed the influence of the anti-vaccination movement and named public figures who have spoken out against vaccination when asked what contributed to not only their HPV vaccine hesitancy, but hesitancy among their family, friends, and community. One participant recalled Jenny McCarthy, an American actress, model, and television personality, publicizing that her child became autistic after receiving a vaccine, “I don’t remember which vaccine it was, but that was- that got me thinking that, hey maybe vaccines are actually not really good for you” (Participant 6, Interview, Sacramento County). Another participant spoke about how she was a part of an online mom’s group and when conversations around vaccination come up, she shared, “there’s a whole string of people with their conspiracy theories that the government is involved, that they don’t feel that they’re safe, that there’s mercury in them, that it causes autism…” (Participant 7, Interview, Nevada County). Participant 5 summarized how vocal and persistent the anti-vaccination movement has been around spreading their message against vaccination:

*“They kind of suck people in easily because their message is so strong and out there. It seems really well organized whereas pro-vaccine seems a little bit more, I don’t know, not as robust…I rarely see a pro-vaccine posting and when I do, there’s like 20 other comments about, ‘Why are you doing that? Why are you posting this pro-vaccine?’ It just gets buried in this anti-vaccine sentiment.”*

Another participant shared a similar response when describing how the anti-vaccination movement has created a culture of intimidation in which individuals are afraid to ask questions about vaccines for fear of backlash from the movement. This participant stated, “I know a lot of people don’t want to voice their opinions or comment their concerns about the vaccines because of anti-vax or whatever or just be made fun of.” (Participant 15, Interview, Yolo County). 

### 3.2. Individual and Group Influences 

All participants described how their own personal perceptions of vaccines, as well as those of their family, community and social networks, have contributed to their HPV vaccine hesitancy. The research team identified three constructs within this determinant: (1) personal beliefs and attitudes about health and prevention; (2) risk perceptions; and (3) personal, family and community members’ experiences with vaccination. 

#### 3.2.1. Personal Beliefs and Attitudes about Health and Prevention 

The participants discussed how their personal attitudes and beliefs about vaccines have contributed to their vaccine hesitancy. The participants shared that they believed that children should develop immunity from vaccine-preventable diseases naturally. As explained by one participant, “Sometimes, protecting them a little too much from getting sick ends up being worse than putting them out there and letting them get sick is basically how I see it” (Participant 8, Focus Group, Merced County). When asked to elaborate on this notion of natural immunity, participants expressed that they believe kids need to be exposed to germs to stimulate their immune system and as a result their immune system will become stronger. Several other participants referenced this natural health philosophy as “letting kids eat dirt”, an expression that suggests that kids need to be exposed to soil, dust, bacteria, germs, etc. As one parent stated, “how I see it, let them eat dirt. It’s okay, it won’t kill them” (Participant 11, Focus Group #1, Merced County). While another one said “…or they [kids] could just eat some dirt and get some immunity to bacteria” (Participant 12, Interview, Sacramento County). Similarly, another participant shared, “… you should just let your kids eat dirt because that, in fact, creates a lot of immunity” (Participant 9, Interview, Nevada County). Among participants, the belief that kids can develop immunity by being exposed to nature was commonly cited as an alternative to vaccination. A participant summarized:

*“I think just getting dirty and letting them be kids and eventually the immunity will increase based on the environment that we actually grew up with. I think that’s just one way kids would be able to grow their immunity by making sure that they’re exposed to any type of environment as possible, I mean outdoors”*

(Participant 10, Interview, Merced County).

In addition to letting kids be exposed to the environment, another participant explained that her mother would always tell her that kids need to also be exposed to other kids, “…human interaction is going to be important for us to develop, you know strong not just muscles but also internally” (Participant 3, Focus Group #1, San Joaquin County).

#### 3.2.2. Risk Perceptions

The participants described how their lack of confidence in vaccine safety and lack of perceived benefits of vaccination contributed to their HPV vaccine hesitancy. The participants shared that they felt that taking the HPV vaccine is a risk as they are concerned with what is inside the vaccine, as well as whether they believed the side effects are worth it. As one participant explains, “Taking a risk of injecting something into your body that may not be necessary and you’re introducing ingredients that are not really organic.” (Participant 6, Interview, Sacramento County). Another participant shares, “Vaccine components are complicated, some consist of heavy metals and regardless, it is still not good for brain cells” (Participant 15, Interview, Placer County). When describing the decision to vaccinate a child, one participant disclosed that the decision weighs heavily on the parent, “Vaccination does not guarantee that we will not get sick…there are a lot of side effects so it should be weighed…. if it is a child then his parents should take responsibility because it happens that children do not tolerate vaccination very well…” (Participant 17, Interview, Placer County). Another participant shared that they thought the HPV vaccine was unnecessary as they did not perceive the benefits of vaccination, “I feel that it’s really unnecessary unless somebody does or they are high risk then they may want to discuss it with their doctor and weigh the pros and cons, but there may be girls out there that are really not at risk and may never get that cancer, so why bother getting the vaccine because it’s not really necessary.” (Participant 6, Interview, Sacramento County). When asked what were the major factors that contributed to HPV vaccine hesitancy, participants cited that safety concerns were one of the main reasons why parents are not getting the HPV vaccine for their children. One focus group participant explained, “I think it comes down to parents being concerned that it’s not safe, those who aren’t getting it” (Participant 5, Focus Group, Nevada County). Another focus group participant echoed similar sentiments, stating “I do think that if people believe these vaccines are safe and effective, they will get them” (Participant 16, Focus Group, Nevada County). 

#### 3.2.3. Personal, Family, or Community Members’ Experiences with Vaccination 

Participants described how their personal, family or community members’ experiences with vaccination have greatly influenced their HPV vaccination decision. This theme was particularly salient among participants who immigrated to the U.S. from another country. These participants shared how their experiences growing up in another country and their culture have influenced their vaccine hesitancy. One participant described, “I noticed here [in the U.S] the difference is that they exaggerate with the babies, and they want to disinfect everything…You know the kids there [Mexico] didn’t even get sick. So, I think the best way for a child to create [immunity] so they can fight against it is to create antibodies so that his own body can fight” (Participant 13, Focus Group #2, San Joaquin County). Slavic participants shared that many members of their community do not believe in vaccinations and these participants recounted vaccine stories that occurred in their home country. One participant shared that her sister had a bad reaction to getting a vaccine, “After about an hour after the injection, her body was having a reaction, her face started swelling, her ears were swelling, and she started turning blue. This happened in Moscow…After that event, no one in my family got vaccinated against anything” (Participant 15, Interview, Yolo County). Another participant shared that the fear of adverse effects is something her community is very concerned with, “…for instance Russia as a whole, many mothers are scared to vaccinate due to the possibility of adverse effects, some may not have had personal experiences [with adverse effects] in their family, but they are scared from hearing about stories and that happen to their children if they give them a vaccine” (Russian Interview 5_23). Another interviewee explained that while some vaccines were acceptable to administer to children to prevent severe diseases, they felt that children in America are being given too many vaccines, “…A lot of young mothers that are here for a while now, we came from Ukraine two years ago, we are used to vaccinating our children because no one wanted their children to be sick with smallpox, mumps, or chickenpox. But here a lot of moms say that NO, in America they administer too many vaccines, it’s very bad for the children, children become stupid, slow, develop autism...” (Participant 16, Interview, Sacramento County). Stories of adverse reactions coupled with the notion that too many vaccines are being administered was stated as the main reasons why participants felt that their community did not vaccinate. One participant stated, “From my Russian friends and people that I know, no one vaccinates.” (Participant 17, Focus Group, Yolo County). Additionally, from that same focus group, another participant concurred, “Here in the U.S. the people I am around are very anti vaccine, any vaccine… [and] yes, it’s mostly neighbors [Russian] or such who advocate against [vaccination].” (Participant 18, Focus Group, Yolo County). 

### 3.3. Vaccine/Vaccination-Specific Influences

The participants discussed how the timing of when the HPV vaccine is recommended to their child from their doctor during a medical appointment and the number of vaccines administered at one time were contributing factors to their vaccine acceptance or resistance.

#### Vaccine Timing

The participants expressed how children are receiving too many vaccines during one medical visit and how the timing of the vaccine conversation contributed to their hesitancy. The participants described how important it is to recommend the HPV vaccine at the right time rather than at every office visit. As one participant states:

*“For comparison, in Moldova, before getting vaccinated, the patient must pass a urine test and a chemical blood test... If one of the indicators in these tests does not correspond to standards or indicates that there is some kind of infection in the body or some aspect of this analysis is bad, then now is not the time to get vaccinated. In America you get vaccinated just because it’s time. And often children with runny noses, with screams, are vaccinated and then there are a lot of such cases that we hear out there. Maybe the vaccination is good but because it was not done at the right time, it played a bad role. So for me it’s so strange”*

(Participant 16, Interview, Yolo County)

In addition to the timing of the vaccine conversation between parents and providers, the participants also discussed their preference of spacing out the vaccines as to not give children too many vaccines at one time. One participant shared “…I did lean towards spacing out the vaccines…we don’t want to give too many vaccines at once. Let your body respond accordingly and then move on to the next one…” (Participant 18, Interview, Sacramento County). A participant summarized the reason they split up the vaccines was because of fear of the reaction:

*“So, when my kids were little, I would always split their vaccinations up because I would call it a martini drink or martini mix because it be so many in one. So, they would get a 3-in-1, so I would always- they were always behind because I would split them up because of reaction that I heard people would have...”*

(Participant 19, Focus Group, Merced County)

### 3.4. Strategies and Recommendations

The participants stressed the importance of providing culturally appropriate, in-language, in-person, easy-to-understand HPV vaccine education that is tailored to the concerns of their community. As expressed by one participant, “I think there needs to be intentional education out there, outreach, and people that speak their language and understand a little bit about the purpose why people need to get those vaccine” (Participant 10, Interview, Merced County). Specifically, for the Russian community, the participants felt that more education is needed. One participant stated, “I think that for the Russian community, we need to be giving more explanation and education for what a vaccine is needed, what are the goals, so people can understand...” When asked about the mode of delivery for intervention strategies, the participants preferred in-person engagement. As expressed by one participant, “Mail, no. Telephone, no. Face to face is best” (Participant #20, Interview, Nevada County). The participants also reminded the research team that language barriers and health literacy were important factors to consider. As identified by one participant, “…you want to make sure that interpretation or translation is also available to people because not everyone can read English or understand big words” (Participant #21, Interview, Yolo County). One participant summarized:

*“…making sure that one’s reading the material that is culturally responsive and you know culturally appropriate because we’re not all the same, so just making sure that whatever materials are being done and whatever’s being distributed, that there is that checking in with the different cultures and making sure that it’s appropriate for their culture…”*

(Participant #22, Focus Group #1, San Joaquin County)

The participants also suggested removing logistical barriers to vaccination by providing the vaccine at community locations. One participant shared, “…the only way I got it [the HPV vaccine] was because there was like this van that would go to low income apartments and they would give free flu shots and stuff like that…My mom went to go get her flu shot and I went with her to translate and that’s when the nurse that was there was talking to my mom and she brought up the HPV vaccine. That’s how I got—I got my first dose there…the only reason I got it was because I was there at that moment.” (Participant #19, Focus Group, Merced County). In addition to clinicians and nurses, the participants also suggested using trusted community leaders as HPV vaccine advocates. One participant shared, “some of our families really do listen to what their leaders in their faith community talk about and then from that also work with teachers because teachers are considered to be trusted sources with information” (Participant #8, Focus Group, Merced County). 

## 4. Discussion

This study broadly examined sources of vaccine hesitancy and described how these sources influenced HPV vaccine decision making among populations who have expressed vaccine hesitancy in counties with low HPV vaccine uptake. To the best of our knowledge, this is the first study to explore HPV vaccine hesitancy from the perspectives of rural, Latino/Hispanic, and Slavic communities. The geographic and ethnic/racial diversity of our participants provides unique insights into these understudied populations that highlight gaps in the existing literature and includes community-recommended strategies for building HPV vaccine confidence. Within these communities, we sought to understand the contextual, individual and group, and vaccine/vaccination-specific influences that the participants discussed as reasons for HPV vaccine hesitancy among themselves and their community. 

Utilizing both focus groups and key informant interviews, we were able to elicit salient factors contributing to HPV vaccine hesitancy in these communities and how these factors influenced their vaccine decision making. Six main themes were identified from the focus groups and interviews: (1) The use of social media in spreading easily accessible negative vaccine-related content regardless of editorial and scientific oversight. (2) The tactics used by the anti-vaccination movement to instill HPV vaccine hesitancy by spreading HPV vaccine misinformation and disinformation, and intimidation. (3) The participants’ vaccination beliefs and attitudes stemming from their personal health philosophies. (4) The participants’ lack of confidence in the safety of the vaccine and how that has contributed to their perceptions of the risk and benefits of HPV vaccination. (5) The vaccine experiences of their family, friends, and community members. (6) The timing of when the HPV vaccine conversation occurs between doctors and parents/patients as well as the number of vaccines administered to children at the same time. 

The participants described the influence of social media on contributing to HPV vaccine hesitancy and how challenging it is to distinguish whether the media content being shared is factual (e.g., “you don’t know if it’s the truth or a lie”) because of how powerful the messaging is. The participants interchangeably discussed social media and the anti-vaccination movement within the same context as social media is one of the main modes the movement utilizes to disseminate their information. The participants shared how social media content on the HPV vaccine consists mostly of HPV vaccine information related to potentially false adverse health effects and claims related to vaccine safety. Supporting our findings, in a prior study assessing the HPV vaccine, Facebook posts from local health agencies and groups within the same geographic region as this study provoked more positive emotion, more negative emotion and more anger than the posts with concerns mostly focused on vaccine safety, risks, and injury [27]. Additionally, prior studies have documented that HPV vaccine misinformation and disinformation spread through social media is a major driver of vaccine hesitancy [27]. Social media usage, particularly among parents, has been growing over the past few decades. A cross-sectional survey was conducted among a group of parents, and the analysis found that nearly all (96%) claimed to use some form of social media; 68% reported using it to obtain health-related information [28]. Even among our racially/ethnically and geographically diverse participants, social media was overwhelmingly mentioned. Promising research shows that HPV vaccine misinformation on social media can be countered with social media messaging that addresses and corrects the misinformation [29,30]. More research is needed on how to develop and implement culturally tailored in-language social media messaging that can combat HPV vaccine misinformation. Interestingly, among our participants who were born in another country, the participants described how the vaccination experiences and health beliefs they held growing up in their native country have contributed to their HPV vaccine hesitancy. This is consistent with a literature review by Daniels et al. that reported refugees, immigrants and migrants had a negative cultural bias towards vaccination because of cultural norms, practices, or beliefs [31]. In another study, 35.3% of surveyed Latina immigrant mothers of 9–12-year-old daughters in Alabama reported HPV vaccine hesitancy despite their physician recommending the vaccine [32]. Tankwanchi et al. suggested that HPV vaccine hesitancy among immigrant populations in the U.S. may be attributed to limited knowledge regarding cervical cancer and HPV vaccination and religion. In our study, while we also found limited knowledge regarding cervical cancer and the HPV vaccine, religion was not cited as a main source of vaccine hesitancy [33]. Within our Hispanic/Latino and Slavic participants, the main reasons described as contributing to their vaccine hesitancy related to their home country were the beliefs that, in the U.S., there is a tendency to over vaccinate children and that not all vaccines are necessary. These participants expressed greater concern for adverse reactions to vaccines and a lower general HPV vaccine knowledge. More research is needed to develop effective HPV vaccination educational materials that would resonate with these communities.

Additionally, the participants explained that personally and within their social networks and community, a major source of HPV vaccine hesitancy is the belief that HPV vaccination is unnecessary because children should develop immunity naturally. The participants repeatedly stated we should “let children eat dirt.” While we know participants are not literally stating that we should let children eat dirt and are merely suggesting that we let children develop immunity naturally through direct exposure to germs and pathogens, it is still alarming that this belief resonated with so many of our participants. Reich describes this dichotomy as parents perceiving the differences in natural and artificial immunity, with vaccination being seen as artificial and inferior to immunity occurring from infection with the disease [34]. Other studies have reported similar findings when exploring parental reasoning for incomplete and/or a lack of childhood vaccinations [35,36,37]. The participants also perceived the vaccine as being high risk with low benefits. The participants described having low confidence in the safety of the vaccine. Consistent with a systematic review of 71 studies on the beliefs around childhood vaccines in the U.S., the participants in our study described that their HPV vaccine hesitancy was influenced by the beliefs that vaccines contain harmful ingredients; natural immunity is better than vaccine acquired immunity; receiving a vaccine can cause illnesses; and that receiving too many vaccines at one time can overwhelm a child’s immune system. [36] 

While fear about the safety and efficacy of the HPV vaccine has been widely reported in the literature [38], our findings regarding a desire for children to develop immunity naturally due to how children are raised in the participants’ native country, coupled with the belief that the U.S. is too fixated on vaccines and cleanliness, adds a unique perspective to the literature. More research is needed to understand the role of social networks and cultural norms among refugee, immigrant, migrant, and rural communities when assessing HPV vaccine uptake. Additionally, educational messaging that provides a clear rationale for vaccination that emphasizes the importance of herd immunization may resonate with these collective communities, as well as an open conversation recognizing that while there are some benefits to natural immunity, vaccines are necessary for the overall health of the population. Interventions targeting these communities should consider including a community strategy as social networks appear to be a significant source of HPV vaccine hesitancy.

### Strengths and Limitations

The results reported here are the views of a specific group of individuals and their experiences are not representative of the entire U.S. population nor of the counties and racial/ethnic groups where we recruited participants, but rather provide a broad overview of sources of HPV vaccine hesitancy among vaccine-hesitant communities that can be used as a starting point for future research inquires seeking generalizability. Additionally, our convenience sample size of seven focus groups and 14 key informant interviews with 39 individuals may be considered modest, thus further limiting the generalizability of our findings. However, prior studies have reported that code saturation for interviews can be reached within the first 12 interviews, with basic themes emerging as early as within six interviews [39,40]. In their study, Guest et al. found that 94% of high frequency codes were identified within the first 6 interviews and 97% were identified after 12 interviews [36]. Chen et al. reported that focus group data saturation, with 84% of themes generated, can be reached within the first three focus groups [41]. Our analysis followed a similar pattern in which we were able to reach data saturation within the first 12 interviews and within the first four focus groups. Our findings were further validated as we found other studies have found similar perspectives on HPV vaccine hesitancy to those we determined. Although we felt our sample size was adequate, additional recruitment of more participants within each strata (e.g., rurality, occupation, race/ethnicity, place of residence, occupation) could strengthen our conclusions and increase the generalizability of our findings. However, generalizability is not the goal of qualitative inquiry, and our intent was to obtain an in-depth understanding and gain insight into the nuances of HPV vaccine hesitancy, including uncovering the reasons behind why and how these communities became hesitant, to develop effective interventions to increase HPV vaccination in these communities. Additionally, our recruitment methods, coupled with the monetary compensation participants received, could have also resulted in a selection bias. We also acknowledge that HPV vaccine hesitancy is influenced by a multitude of factors such as social demographics, adolescent gender, and health care usage, which were not assessed in this study. The inclusion of prevalence and characteristics of HPV vaccine hesitancy is beyond the scope of this qualitative study; thus, a quantitative follow-up study that can assess statistically significant determinants of HPV vaccine hesitancy is needed to validate our findings. 

Despite these limitations, the strengths of this study include purposeful recruitment of individuals from underrepresented communities (e.g., Slavic, Latino, and rural) and being able to solicit in-depth responses regarding their views on vaccinations. This study can be used to further research determining factors associated with HPV vaccine hesitancy in these communities, which will result in effective interventions to combat HPV vaccine hesitancy within these communities.

## 5. Conclusions

Among our diverse group of participants that included rural and racial/ethnic diverse foreign-born individuals, we found that the sources of HPV vaccine hesitancy existed across all three determinants of the Vaccine Hesitancy Determinant Matrix. While our findings are consistent with prior studies, our study contributes to the growing literature on understanding local sources of HPV vaccine hesitancy as the factors that influence vaccine hesitancy are unique to each community, especially among Hispanic/Latino, Slavic, and rural communities. This qualitative study allowed participants to describe in their own words and in their language of choice what those factors were and how these have shaped their beliefs and, ultimately, their HPV vaccine decision-making process. Our findings can help inform future interventions to increase HPV vaccine uptake by addressing factors most likely contributing to HPV vaccine hesitancy in these communities.

## Figures and Tables

**Table 1:** General interview/focus group vaccine hesitancy determinant matrix.

Determinant	Construct	Interview/Focus Group Question
Contextual Influences	Communication and media environment	Who do you trust the most for information about vaccines and why?
		Who do you trust the least and why?
		Where do you go for trusted information about vaccinations and why?
		Do you share information related to vaccination within your own social media network? Probe: What type of information do you share and on what platform?
	Influential leaders, gatekeepers, and anti- or pro-vaccination lobbies	Are you practicing [religion] if so which religion? Does ____ your place of worship, have any recommendations/thoughts on vaccines?
		What is your religion/philosophy/culture’s stance on vaccination? Probe: If positive/negative: Which vaccines? What is the reason [religion, philosophy, or culture]? Probe: Has your community in the past refused to accept certain vaccines which ones and why?
		What have leaders (religious, political, teachers, health care workers) in your community said about childhood vaccinations? How about the HPV vaccine? Probe: [If advice was given] Did you follow this advice?
	Historical influences	Do you remember any events in the past that would discourage you from getting a vaccine for yourself or your child/children (if you have kids)? Probe: Have reports you heard/read in the media/on social media made you reconsider the choice to have to yourself or your child vaccinated? Probe: If yes, do you remember the source of that information? Who posted that information?
	Politics or policies	Do you trust (or distrust) that our government is making decisions in your best interest with respect to what vaccines are available? Probe: What about vaccines that are required (list these: if needed) Probe: Do you disagree with the choice of vaccine or vaccination recommendation by the government?
	Geographic barriers	[Only if they have kids] Has distance, clinic hours, time needed to get to clinic or clinic wait time and/or vaccine costs prevented you from getting your child immunized? Probe: If yes, which ones were the biggest factors?
Individual and Group Influences	Immunization as a social norm vs. not needed/harmful	Do you think that most parents have their children vaccinated with all the recommended vaccines? What about the HPV vaccine?
	Health system and providers’ trust and personal experience	Do you feel able to openly discuss your concerns about vaccines with your doctor? Probe: Do you trust the information you receive about vaccines your provider? Probe: Do you feel that your health care provider cares about what is best for your child?
Vaccine or Vaccination Specific Issues	Introduction of new vaccine	Are you familiar with the HPV vaccine? If yes, no what do you know about it?
	Risk/benefit	Has your child been vaccinated against HPV, yes or no? Probe: Concerns
		Do you believe vaccines are safe for yourself, for your child [remember if they have kids or not], for those in your community?
	Vaccination schedule	Have you ever delayed vaccinating your child with a newly introduced/recommended vaccine, if so why?

**Table 2:** Description of focus group and key informant participants.

Residential County	Method (I/FG) *	Conduct Language	Population	Gender	Number and Type of Participants
El Dorado	I	English	Rural—non-Hispanic White	Female	1 (community)
El Dorado	I	English	Rural—non-Hispanic White	Female	1 (parent)
Nevada	I	English	Rural—non-Hispanic White	Male	1 (health professional/parent)
Nevada	I	English	Rural—non-Hispanic White	Female	1 (health professional)
Nevada	FG	English	Rural—non-Hispanic White	2—Female 1—Male	3 (parents/health professionals)
Nevada	I	Spanish	Latino/Hispanic	Female	1 (community health worker/parent)
Merced	I	English	Hmong	Male	1 (parent)
Merced	FG	English	Hispanic, Hmong, and Rural	All female	5 (parents and school staff)
Merced	FG	English	Hispanic, Hmong, and Rural	All female	4 (parents and school staff)
Sacramento	I	English	non—Hispanic white	Female	1 (community)
Sacramento	I	English	All	Female	1 (parent/cancer organization)
Sacramento	I	English	All	Female	1 (parent/cancer organization)
Sacramento	I	Russian	Slavic	Female	1 (parent)
Sacramento	I	Russian	Slavic	Female	1 (parent)
Sacramento	FG	Russian	Slavic	Both female	2 (parents)
San Joaquin	FG	English	Latino/Hispanic	2—Female 1—Male	3 (parents and community)
San Joaquin	FG	English	Latino/Hispanic	4—Female 1—Male	5 (parents and community)
Placer	I	Russian	Slavic	Female	1 (parent)
Placer	I	Russian	Slavic	Female	1 (parent)
Yolo	I	English	Latino/Hispanic	Female	1 (parent)
Yolo	FG	English	All	All female	3 (parents and community)

* I = Interview and FG = Focus Group.

## Data Availability

The deidentified data underlying the results presented in this study may be made available upon reasonable request from the corresponding author, Julie HT Dang (jtdang@ucdavis.edu).
